# Prevalence of virulence genes in *Enterococcus* species isolated from companion animals and livestock

**DOI:** 10.4102/ojvr.v85i1.1583

**Published:** 2018-06-27

**Authors:** Shirwin Pillay, Oliver T. Zishiri, Matthew A. Adeleke

**Affiliations:** 1 School of Life Sciences, College of Agriculture, Engineering and Science, University of KwaZulu-Natal, South Africa

## Abstract

*Enterococcus* species have developed from being commensal bacteria to leading pathogens that cause infections in humans and animals. The gastrointestinal tract of mammals is the normal habitat of these species. Virulence factors are proteins that are produced by the bacterium which are used to enhance their pathogenicity. The objectives of this study were to isolate *Enterococcus* spp. from livestock and companion animals, differentiate between the different sub-species and detect the presence of important virulence genes. Rectal and saliva swabs were collected from dogs and cats, whereas only rectal swabs were collected from cattle and cloacal swabs from chickens. Presumptive *Enterococcus* was selected using Bile Esculin Azide (BEA) agar, and *Enterococcus* species were confirmed using the polymerase chain reaction (PCR) by amplifying the *tuf* gene. In order to differentiate between *E. faecalis* and *E. faecium,* a multiplex PCR was used to detect the *SodA* gene. The genes responsible for gelatinase production (*gelE*) and for conjugation (*ccf*) were also detected using PCR. Out of 211 animal swabs, 182 (86%) were positive for the *tuf* gene. Overall, there were 55 isolates of *E. faecalis* (30%) compared to 22 isolates of *E. faecium* (12%). The virulence genes had a prevalence of 52% and 36% for *gelE* and *ccf,* respectively, in all animal hosts. The results demonstrated that chicken cloacal samples had the highest prevalence for *E. faecalis, gelE* and *ccf* genes compared to all the other isolates detected from other animal hosts. The results also demonstrated a statistically significant (*p* < 0.05) association between the prevalence of virulence genes (*gelE* and *ccf*) and animal species from which *Enterococcus* spp. was isolated. We provided evidence that healthy livestock and companion animals can harbour pathogenic *Enterococcus* that can be transferred via the food chain as well as through close association such as petting and licking of humans. This study partially demonstrated that *Enterococci* spp. are capable of evolving from being simple commensal bacteria to becoming pathogens that cause infection in humans and animals through the acquisition of virulence factors through mobile genetic elements.

## Introduction

*Enterococcus* species are a diverse group of Gram-positive, facultative anaerobic bacteria that have a wide adaptability to withstand harsh conditions like temperature, pH, hyperosmolarity and prolonged desiccation (Ali et al. 2014; Lebreton, Willems & Gilmore 2014; Moraes et al. 2012). *Enterococcus* species contain the group D cell wall antigen, which is associated with the cytoplasmic membrane. Hence, they were initially classified as Group D *Streptococci* (Teixeira & Merquior 2013). Cetinkaya, Falk and Mayhall (2000) reported that *Enterococcus* was suggested to be a genus on its own and not a part of the *Streptococcus* genus. This was proposed because of DNA–DNA and DNA–rRNA hybridisation revealing that species such as *Streptococcus faecalis* (now *Enterococcus faecalis*) and *Streptococcus faecium* (now *Enterococcus faecium*) were relatively distantly related to non-Enterococcal *Streptococci*. Molecular DNA studies demonstrated that *Enterococcus* should be classified as its own genus as there are significant differences between *Enterococcus* and *Streptococcus* (Byappanahalli et al. 2012). Successful detection of *Enterococcus* species can be achieved by simply detecting the *tuf* gene (encoding elongation factor) using the polymerase chain reaction (PCR). The *tuf* gene encodes the elongation factor EF-Tu and is involved in peptide chain formation. This gene is a highly evolutionarily conserved part of the core genome, and is more discriminative than the 16S rRNA gene for identifying organisms belonging to the *Enterococcus* genus (Li et al. 2012). In order to differentiate the species of *Enterococcus*, the *SodA* gene, which encodes a manganese-dependent superoxide dismutase, can be used. This gene is more discriminative compared to the 16S rRNA in differentiating closely related species. Previous studies have shown that fragments of the *SodA* gene of *Enterococcus casselifalvus* and *Enterococcus flavescens* were 99.5% identical and therefore should be considered the same species (Poyart, Quesnes & Trieu-cuot 2000).

The ability of *Enterococcus* spp. to cause infections has been associated with the species’ intrinsic ruggedness (Santagati, Campanile & Stefani 2012). This trait allows the enterococcal species to persevere in hospital environments and allows the microorganism to withstand a variety of host defences such as the innate immune system. The innate immune defence is an essential first step in combatting infectious disease. To establish infections, pathogens evolved strategies to overcome this defence. Innate immunity consists of the humoral components such as the complement system and cellular components including polymorphonuclear leukocytes, macrophages, mast cells, basophils, eosinophils and dendritic cells. Resistance mechanisms against the innate immune system which enable this commensal organism to become pathogenic are widely unknown. The genetic flexibility of *Enterococci* is an important feature. They are equipped with many antibiotic resistance and virulence genes that can be attained and transferred (Chaje˛cka-Wierzchowska, Zadernowska & Łaniewska-Trokenheim 2017; Fisher & Phillips 2009).

Some virulence factors are regulated by virulence coding genes present on plasmids or in specific regions on the genome known as pathogenicity islands (PAI). The PAI contain multiple pathogenicity factors, for example, the enterococcal surface protein (ESP). The ESP is responsible for an increased biofilm formation and colonisation potential. An important virulence factor is an extracellular active gelatinase (GelE). This extracellular zinc-metalloprotease is part of a protection mechanism against the host response (Waters et al. 2003). Gelatinase is essential for resistance to several key components of the host innate immune defence system including antimicrobial peptides preventing damage to the pathogen (Potempa & Pike 2009). The enzyme causes cleavage and degradation of host proteins like LL-37, fibrinogen, fibrin and collage (Scheb-Wetzel et al. 2014). Comerlato et al. (2013) reported that GelE-positive strains of *E. faecalis* had an increasing potential to establish a biofilm. These findings underline the importance of GelE for host colonisation and virulence (Scheb-Wetzel et al. 2014).

According to Eaton and Gasson (2001), virulence genes are transferred by a highly efficient transfer mechanism. Species that lack plasmids (recipients) excrete sex pheromones which induce a mating response in *Enterococcus* that have a certain plasmid (donor), causing an aggregation of recipients and donor cells (Clewell & Weaver 1989). Hirt et al. (2005) reported that, *E. faecalis*’ sex pheromone plasmids are one of the most efficient conjugative plasmid transfer systems known in bacteria. Sex pheromones in *Enterococcus* are encoded on the *cob, cpd* and *ccf* genes. Studies have demonstrated that *Enterococcus* strains that possessed and expressed virulence factors caused a more serious infection than strains that lacked virulence factors (Chaje˛cka-Wierzchowska et al. 2017). The process of infection involves specific steps such as colonisation, adhesion, tissue invasion and a defence mechanism such as resistance (Flores-Meireles et al. 2015; Upandhyaya, Ravikumar & Umapathy 2009). It is important to note that in order to be pathogenic, antibiotic resistance genes have to be accompanied by virulence factors and genes (Heidari et al. 2016).

Many *Enterococci* spp. are mostly associated with the intestines of domestic animals and humans (Wurster, Saavedra & Gilmore 2016). They play a fundamental role of commensal bacteria found in the microbial consortia in the gut, aiding in the degradation and digestion of food as well as other metabolic pathways (Hammerum 2012; Santagati et al. 2012). *Enterococcus faecalis* and *E. faecium* are the most prevalent enterococcal isolates found within the human gastrointestinal tract (Fisher & Phillips 2009). *Enterococcus species’* wide adaptability allows the microorganisms to colonise various habitats from hospitals to the human and animal guts and to the natural environment (Lebreton et al. 2014). When these organisms are localised outside the gut, they are considered as faecal pollution indicators. With regard to the human body, they are considered as human pathogens (Santagati et al. 2012). In recent years, there has been substantial progress in the detection of virulence factors in Enterococci of clinical origin. This has therefore made the detection of virulence genes in strains isolated from food possible. Zoonoses are diseases or infections that are transferred from animals to humans and humans to animals either directly or indirectly (WHO 2017). Foodborne zoonoses are a major public health concern worldwide. The African region has the highest incidence and death rates because of these diseases (WHO 2015).

It is therefore important to pay close attention to animals that are closely associated with humans such as companion animals and livestock. Pathogenic bacteria can be transferred through animal saliva, open wounds and contaminated meat from livestock (Schjørring & Krogfelt 2011). Furthermore, human and companion animal contact because of sharing of common environments will also exacerbate cross transfer of pathogenic bacteria. Once the bacteria are in the intestines of humans, they may colonise and persist or be present for a short time, which may be a sufficient time for virulence and resistance genes to be transferred to other strains, providing them with ‘weapons’ to cause infections (Nilsson 2012). To our knowledge, there is a paucity of research conducted on the detection and differentiation of *Enterococcus* species in companion animals and livestock in South Africa, let alone KwaZulu-Natal Province. Against this background, the aim of the study was to investigate the prevalence of *Enterococcus* spp. in livestock and companion animals in the Durban Metropolitan area. Furthermore, confirmed *Enterococcus* spp. were differentiated based on the presence of *gelE* and *ccf* virulence genes.

## Materials and methods

### Sample collection

A total of 211 samples (Table 1) were collected, processed and analysed for this study. Sterile swabs were used to randomly collect 70 rectal and mouth swabs from 36 dogs and 34 cats, among those that were treated at a veterinary clinic in Durban. The pets that are included in this study live in households in Durban. Seven race horse faecal samples were also included in the study. Thirty-four cattle rectal samples were collected from a herd, and 30 chicken cloacal samples were collected from a chicken farm in Pietermaritzburg (KZN). Samples were collected between October 2016 and February 2017. Upon collection, the swabs were placed in 5 mL buffered peptone water (BPW) (Merck) for enrichment in 15 mL centrifuge tubes and transported to the University of KwaZulu-Natal Westville campus for further processing and analyses.

**TABLE 1:** Distribution of samples based on animal species and site of swab collection.

Animal species	Site of swab collection	Number of animals sampled
Dogs	Rectal	36
	Mouth	36
Cats	Rectal	34
	Mouth	34
Cattle	Rectal	34
Chickens	Cloacal	30
Horses	Faecal	7

### Identification and confirmation of *Enterococcus* species

Upon arrival at the laboratory, the samples were incubated at 37 °C for 24 hours. The samples were further enriched in Trypticase Soy Broth (TSB) by adding 1 mL of the BPW to 5 mL TSB and incubated at 37 °C for 24 h. Bile Esculin Azide (BEA) agar was used to presumptively identify *Enterococcus* species. A loopful of sample was streaked onto the BEA agar plate and incubated at 37 °C for 24 h. The detection of *Enterococcus* species is based on the hydrolysis of esculin in the media into glucose and esculetin. The esculetin reacts with a ferric iron salt to produce a phenolic iron complex, which turns the medium dark brown or black. Bile Esculin Azide agar contains ingredients such as bile salts and sodium azide to inhibit the growth of other Gram-positive and Gram-negative organisms, respectively. Single colonies that turned the media black were selected and were added to 5 mL of TSB. This was incubated as previously described for DNA extraction and PCR. Deoxyribonucleic acid extraction was performed according to Ruiz-barba, Maldonado and Jiménez-díaz (2005). The concentration and quality of the isolated DNA were checked with the use of the Thermo-Scientific Nanodrop 2000, UV-VIS Spectrophotometer (Wilmington, Delaware, USA). A 25.0 *µ*L PCR was used to amplify the *tuf* gene (112 bp) in order to confirm enterococcal species using primers (Table 2). The reaction mixture contained 12.5 *µ*L Thermo-Scientific master mix, 1 pmol (1.0 *µ*L of 10.0 *µ*M) of each primer, 5.5 *µ*L of sterile H_2_O and 5.0 *µ*L of extracted DNA. As positive and negative controls, known DNA from *E. faecalis* and sterile water were used, respectively. The thermocycler conditions were as follows: initial denaturation at 94 °C for 4 min followed by 34 cycles of denaturation at 94 °C for 1 min, annealing at 53 °C for 1 min, extension at 72 °C for 1 min followed by the final extension at 72 °C for 5 min. Thereafter, the PCR products were subjected to gel electrophoresis using a 1.5% agarose gel stained with 10 mg/mL ethidium bromide for 40 min at 80 V. A 100 bp ladder was used to determine the size of the product amplified. Products were visualised under UV light in the BIO-RAD, ChemiDoc™ MP Imaging System.

**TABLE 2:** Target genes, oligonucleotide primer sequences, amplicon sizes and annealing temperatures used for the detection of *Enterococcus faecalis* and *Enterococcus faecium.*

Gene	Primer	Sequence	Product size	Ta (°C)	Reference
***tuf***	Forward	5′-TACTGACAAACCATTCATGATG-3′	112 bp	53	(Ke et al. 1999)
	Reverse	5′-AACTTCGTCACCAACGCGAAC-3′			
***SodA - E. faecalis***	Forward	5′-ACTTATGTGACTAACTTAACC-3′	360 bp	55	(Jackson, Fedorka-Cray & Barrett 2004)
	Reverse	5′-TAATGGTGAATCTTGGTTTGG-3′			
***SodA - E. faecium***	Forward	5′-GAAAAAACAATAGAAGAATTAT-3′	215 bp	48	(Jackson et al. 2004)
	Reverse	5′-TGCTTTTTTGAATTCTTCTTTA-3′			
***gelE***	Forward	5′-ACC CCG TAT CAT TGG TTT-3′	419 bp	55	(Eaton & Gasson 2001)
	Reverse	5′-ACG CAT TGC TTT TCC ATC-3′			
***ccf***	Forward	5′-GGG AAT TGA GTA GTG AAG AAG-3′	543 bp	52.5	(Eaton & Gasson 2001)
	Reverse	5′-AGC CGC TAA AAT CGG TAA AAT-3′			

Ta, annealing temperature.

### Identification of *E. faecalis* and *E. faecium*

A 25.0 *µ*L multiplex PCR was used to detect the *sodA* gene in positive *Enterococcus* species. The primer sequence (Table 2) used to detect the *sodA* gene, of 210 bp and 360 bp for *E. faecalis* and *E. faecium,* respectively, is depicted in Table 2. The reaction mixture contained 12.5 *µ*L of the 10X master mix, 4.5 *µ*L of sterile water, 1 pmol of each primer and 4.0 *µ*L of the extracted DNA. Four microliters (4.0 *µ*L) of known DNA was used as a positive control and 4.0 *µ*L of sterile water was used as a negative control. The thermocycler conditions were as follows: initial denaturation at 95 °C for 3 min, followed by 34 cycles of denaturation of 95 °C for 30 s, two annealing steps at 48 °C and 55 °C for 30 s each, extension at 72 °C for 1 min and a final extension step at 72 °C for 7 min. To visualise PCR products, electrophoresis was run for 60 min at 80 V using a 1.5% agarose gel which was stained with 10 mg/mL ethidium bromide. The size of PCR products was visualised under UV light in the BIO-RAD, ChemiDoc™ MP Imaging System.

### Detection of *gelE* and *ccf* virulence genes

The *gelE* and the *ccf* genes were amplified using PCR in a 25.0 *µ*L reaction, using previously described primers as indicated in Table 2. A 25.0 *µ*L reaction was used for each gene. The reaction mixture contained 12.5 *µ*L of the 10X master mix by Thermo-Scientific, 5.5 *µ*L of sterile water, 1 pmol of each primer (Table 2) and 5.0 *µ*L of the extracted DNA. For negative control PCR, 5.0 *µ*L of sterile water was used. The thermocycler conditions were as follows for amplification of the *gelE* gene: 34 cycles of denaturation of 94.0 °C for 1 min, annealing steps at 50.0 °C for 1 min, extension at 72.0 °C for 1 min and a final extension step at 72.0 °C for 10 min. For amplification of the *ccf* gene, the conditions were initial denaturation at 94.0 °C for 4 min followed by 35 cycles of denaturation at 94.0 °C for 1 min, annealing at 51.3 °C for 1 min, extension at 72.0 °C for 1 min followed by the final extension at 72.0 °C for 5 min.

PCR products were subjected to gel electrophoresis using a 1.5% agarose gel for 40 min at 80 V which was stained with 10 mg/mL ethidium bromide. A 100 bp ladder was used to determine the size of the product amplified. Products were visualised under UV light in the BIO-RAD, ChemiDoc™ MP Imaging System.

### Statistical analysis

Genes for species identification and virulence were statistically analysed using IBM SPSS statistics (version 24). Chi-square tests were used to test the significance of the prevalence of genes detected from the different sites and species of animals. A binary logistic regression analysis was applied to evaluate the relationship between genes detected in companion animals and livestock. The model included the presence and absence of each gene used to detect species and virulence factor. The data were coded as 1 or 0 if the gene was present or absent, respectively. Associations were considered significant when *p* < 0.05.

### Ethical considerations

Animal studies have been approved by the appropriate ethics committee of the University of KwaZulu-Natal (Reference: AREC/040/016M); therefore, they have been performed in accordance with the ethical standards laid down in the 1964 Declaration of Helsinki and its later amendments.

## Results

**FIGURE 1:**
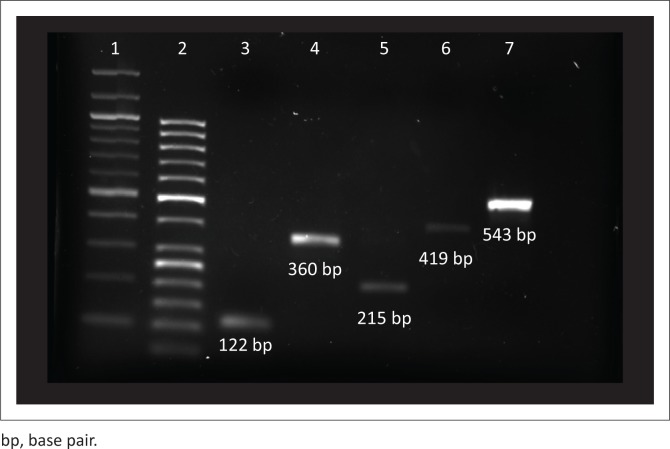
Represents the successful genomic DNA amplification of the *tuf* gene (112 bp), from dog rectal sample 6 in lane 3, *SodA* gene for *Enterococcus faecalis* from dog saliva sample 25 (360 bp) and *Enterococcus faecium* from chicken cloacal sample 20 (215 bp) in lane 4 and lane 5, respectively, *gelE* (419 bp) in lane 6 and the *ccf* gene (543 bp) in lane 7 from chicken cloacal sample 10. Lane 1 and lane 2 are 100 bp and 50 bp molecular weight markers, respectively.

The gel image shown in Figure 1 represents the target genes that were amplified in this study.

Out of a total of 211 animal swabs examined to detect the presence of *Enterococcus* species, 86% (182) of the samples were positive for the *tuf* gene.

**FIGURE 2:**
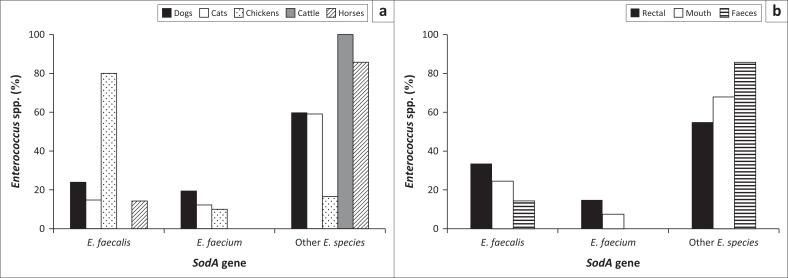
(a) Species within animal sources and (b) species within site of collection – prevalence of *Enterococcus* spp. detected in each animal species and within each site of collection.

The results in Figure 2a and b depict the prevalence of the *E. faecali*s and *E. faecium* and other *Enterococcus* spp. in each animal species and the site of isolation, respectively. *Enterococcus faecalis* was mostly present in chicken cloacal samples (80%), while cattle rectal samples had no incidence of *E. faecalis* or *E. faecium*. Figure 2b shows that rectal and cloacal samples have a higher incidence of *E. faecalis* compared to *E. faecium.* Overall, there was a higher prevalence of undifferentiated *Enterococcus* spp.

**FIGURE 3:**
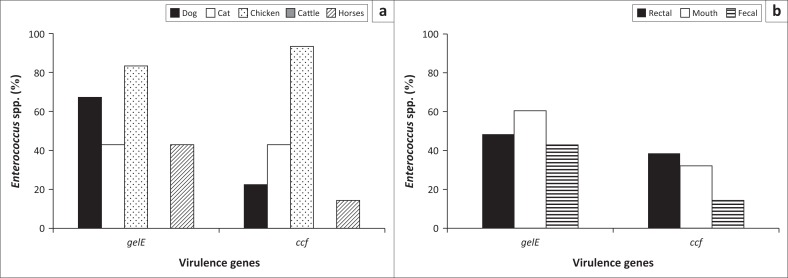
(a) Virulence genes within animal species and (b) virulence genes within site of collection – prevalence of virulence genes detected in each animal species and within each site of collection.

**FIGURE 4:**
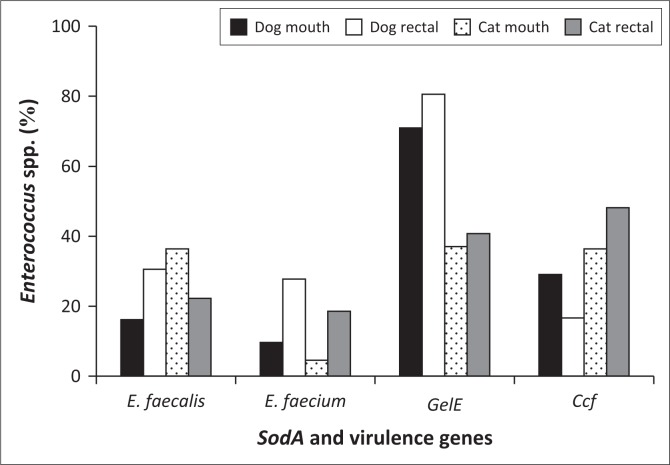
Prevalence of *Enterococcus faecalis, Enterococcus faecium* and virulence genes *gelE* and *ccf* in companion animals.

The prevalence of the virulence genes, *gelE and ccf,* in each animal species and the site of isolation, respectively, is shown in Figure 3a and b, respectively. Chicken samples had the highest incidence of virulence genes, while there was no incidence of the two virulence genes in cattle samples. Dogs had the second highest prevalence of *gelE* gene, while cats were next to chickens with respect to the prevalence of *ccf* gene. Figure 3b shows that *gelE* was common in saliva of the animals, while the *ccf* gene was commonly found in rectal samples. The *Enterococcus* species and virulence genes present on each site of swab collection from dogs and cats are presented in Figure 4.

**FIGURE 5:**
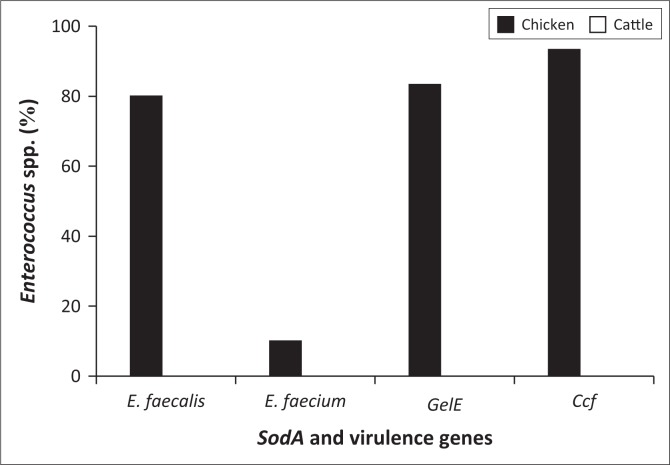
Prevalence of *Enterococcus faecalis, Enterococcus faecium* and virulence genes *gelE* and *ccf* in livestock.

Figure 5 indicates that *E. faecalis, E. faecium* and the two virulence genes were not detected in the cattle rectal samples. However, there was a high incidence of *E. faecalis, gelE* and the *ccf* gene in the chicken rectal samples.

**FIGURE 6:**
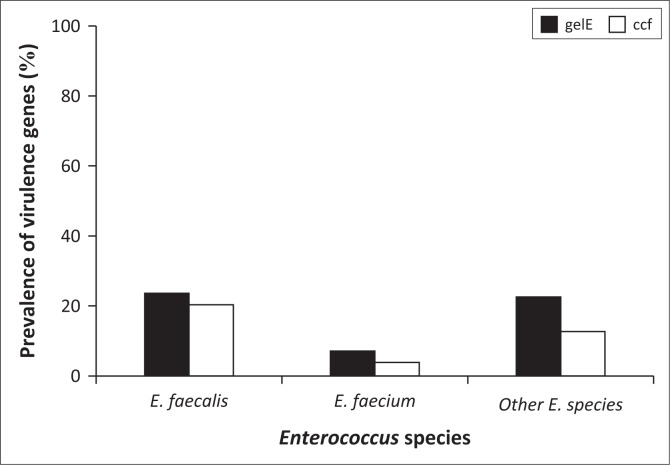
Prevalence of each virulence gene detected within each Enterococcal species.

Figure 6 indicates that *E. faecalis* had a higher incidence than *E. faecium* and the unknown enterococcal species as per this study. Figure 6 also indicates that there was a higher prevalence of *E. faecalis* of the two species. *Enterococcus faecalis* had a higher prevalence of both the *ccf* gene and the *gelE* gene. Therefore, in this study, *E. faecalis* was the more common potentially pathogenic species.

Results of chi-square test, likelihood ratio and Fischer’s exact test for species-specific and virulence genes are presented in Table 3. The prevalence of *E. faecalis* from source was statistically significant (*p* < 0.05) compared to *E. faecium* which was statistically insignificant (*p* > 0.05). Both virulence genes were very highly significant (*p* < 0.001) from the source. With regard to the site of sampling, genes used to identify species and for virulence were statically insignificant (*p* > 0.05).

**TABLE 3:** Chi-square test, likelihood ratio and Fischer’s exact test for target *Enterococcus* spp. and virulence genes.

Source of variation	Statistical test	*E. faecalis*	*E. faecium*	*gelE*	*ccf*
**Animal species**	Chi-square test	0.001	0.076	0.001	0.001
	Likelihood ratio	0.001	0.015	0.001	0.001
	Fischer’s exact test	0.001	0.067	0.001	0.001
**Site of swab collection**	Chi-square test	0.313	0.246	0.307	0.346
	Likelihood ratio	0.291	0.154	0.305	0.310
	Fischer’s exact test	0.404	0.374	0.302	0.392

The logistic regression analysis represented in Table 4 was statistically insignificant (*p* > 0.05) for all the genes except for *E. faecium* and the gelatinase gene (*gelE*) in animal species. B represents the regression coefficient and defines the rate of change of one variable as a function of changes in the other. It is also the slope of the regression line. Taking the exponential of B produced Exp(B), also known as odds ratio, which determines whether there is association between the categorical variables and sources of variation tested at 95% confidence interval.

**TABLE 4:** Null model output from binary logistic regression showing association of virulence genes detected in *Enterococcus* spp. between companion animals and livestock.

Species or gene	Source of variation	B	SE	*p*	Exp(B)	95% CI for EXP(B)	
						Lower	Upper
***E. faecalis***	Animal species	−0.049	0.140	0.725	0.952	0.724	1.253
	Site	−0.492	0.324	0.128	0.611	0.324	1.152
***E. faecium***	Animal species	−0.872	0.281	0.002	0.418	0.241	0.725
	Site	−1.383	0.600	0.021	0.251	0.077	0.813
***gelE***	Animal species	−0.515	0.134	0.001	0.597	0.460	0.776
	Site	0.208	0.287	0.468	1.231	0.702	2.161
***ccf***	Animal species	0.006	0.131	0.964	1.006	0.778	1.301
	Site	−0.400	0.295	0.176	0.671	0.376	1.196

Site, site of swab collection; B, parameter estimates (log-odds) known as coefficient for the constant in the null model; SE, standard error around the parameter estimates; *p* = probability indicating level of significance; Exp(B), exponential of B (also called ‘odds ratio’) for the independent variable Xi; 95% CI, 95% confidence interval.

## Discussion

Animal faeces that contains virulent *Enterococcus* species poses a major public health threat. These bacterial strains can be transmitted to closely related humans through the eating of contaminated food which allows the spread and perseverance of bacteria in the general population and environment (Daniel et al. 2015). In this study, we evaluated *Enterococcus* species isolated from companion animals and livestock as well as the presence of virulence genes. A high incidence of *Enterococcus* species was observed (86%). This is in concordance with several studies from around the world as *Enterococcus* species are commensal organisms that inhabit the gastrointestinal tract of animals. *Enterococci* spp. have been previously reported in high incidence in several reports from South Africa (Iweriebor, Obi & Okoh 2015), Tunisia (Said et al. 2017), China (Liu et al. 2012), Nigeria (Anyanwu & Obetta 2015), Korea (Bang et al. 2017; Kwon et al. 2012), Turkey (Gökmen et al. 2017) and Australia (Barlow et al. 2017). Comparative studies are currently not feasible because there is a paucity of similar studies on *Enterococcus* species from livestock animals and companion animals under South African conditions. However, Iweriebor et al. (2015) reported the detection of *Enterococcus* species as well as the gelatinase gene in piggeries in the Eastern Cape Province of South Africa and concluded that *Enterococcus* spp. from pigs must be treated with the highest caution because they may be reservoirs for virulence and antibiotic resistance genes. Molale and Bezuidenhout (2016) provided evidence on the virulence determinants in *Enterococcus* spp. from surface water systems in South Africa. These sources can allow the spread of resistance and virulent bacteria (Carvalho et al. 2014). When Enterococci isolates were analysed by species, *E. faecalis* was found overall to be the prevalent species. The results from this study were similar to studies, where *E. faecali*s was reported the most prevalent species (Yildiz & Turkyilmaz 2015).

In chickens, *E. faecalis* (80%) was highly present compared to *E. faecium* (10%). Aslam et al. (2012) reported a similar result where overall *E. faecalis* was the most commonly identified species. Yılmaz et al. (2016) reported that chicken meat samples harboured a higher incidence of *E. faecalis* (98%) than *E. faecium* (~1%). However, a study conducted in Nigeria by Ngbede et al. (2017) showed that *E. faecium* was the predominant (49%) species in chicken faeces. A similar result was also observed by Ali et al. (2014) and Ünal, As˛kar and Yildirim (2017), with a prevalence of 66.0% and 33.6%, respectively. In these studies, *E. faecalis* was the second most prevalent species in chicken samples.

The reason as to why we did not detect *E. faecalis* and *E. faecium* in cattle as well the virulence genes is unclear as this was not observed in other studies. Yılmaz et al. (2016) detected a high prevalence of *E. faecalis* (100%) in beef samples. Barlow et al. (2017) detected 6.4% and 8.0% for *E. faecalis and E. faecium* in the order listed from cattle faecal samples. S^ˆ^eputiene˙ et al. (2012) showed that *E. faecium* strains did not carry the *gelE* gene but only *E. faecalis*. Ngbede et al. (2017) showed that 23.8% of cattle rectal species were positive for the *gelE* gene. However, Aslam et al. (2012) reported that *E. hirae* was a predominant species in beef samples. The lack of virulence genes from healthy animals therefore requires further investigation.

Molecular screening of genes which encode virulence factors revealed that the sex pheromone gene, *ccf*, was prevalent in *E. faecium*. According to Eaton and Gasson (2001), the sex pheromone genes, *ccf,* as well as *cob* and *cbp* were not detected in *E. faecium* strains. The incidence of the *gelE* gene was the most predominant virulence factor. As previously alluded, the *gelE* gene encodes the gelatinase enzyme that is responsible for the hydrolysis of haemoglobin, collagen, casein, insulin, fibrinogen, gelatin and other proteins (Upandhyaya et al. 2009). Regardless of the fact that the *gelE* gene was highly prevalent, it is not indicative of the production of gelatinase. It has been suggested that there are other genes which are associated with the expression of gelatinase (Lindenstrauß et al. 2011).

People who have close relationships with household pets produce conditions for bacteria to be transferred to and from their pets through licking, petting, feeding and cleaning. This is a potential threat to the health of human beings if thorough personal hygiene and routine animal and home disinfection is not practised. *Enterococcus faecalis* was the most prevalent *Enterococcus* species in dogs and *E. faecium* was the most prevalent species in cats. Issepi et al. (2015) reported that *E. faecium* and *E. faecalis* were the most frequently isolated species from faeces of dogs and cats. It was also reported that strains of *Enterococcus* had a high incidence of the gelatinase gene as evidenced in our study. Kataoka et al. (2014) and Ossiprandi and Zerbini (2015) reported similar results whereby *E. faecalis* was most prevalent in dogs. Iseppi et al. (2015) explained that we should not exclude the possibility that *Enterococcus* strains had silent virulence genes as well and that it is known that environmental signals can play a vital role in gene expression, hence influencing pathogenicity. According to Kataoka et al. (2014), animals are generally not affected by enterococcal infections; however, they act as a reservoir for pathogenic strains. Therefore, the detection of virulence factors of Enterococci in animals is crucial.

There is a statistically significant (*p* < 0.05) relationship among *E. faecalis* detected from the different animal species used in this study. This indicates that the *E. faecalis* detected are dependent on animal species. For the virulence genes (*gelE* and *ccf*) identified in the animal species, their prevalence depends on each other because the probability for the chi-square is very highly significant showing a statistically significant (*p* < 0.05) association between the occurrence and the absence of *gelE* and *ccf*. Non-significance (*p* > 0.05) of virulence genes shows that occurrence of these genes is completely independent of site of sampling. Furthermore, the probability of detecting *E. faecalis, gelE* and *ccf* in different animal species sampled is 0.1%. The probability of odds for detecting *E. faecium* decreased by 58.2% and 74.9% in animal species and site of swab collection, respectively, while that of *gelE* would drop by 40.3%.

## Conclusion

Our study detected the prevalence of the two most important enterococcal species (*E. faecalis* and *E. faecium*) from companion animals and livestock. We also detected virulence genes encoding gelatinase and a pheromone that induces conjugation, that is, *gelE* and *ccf*, respectively. In addition, the results also indicated that there was a strong association between the prevalence of virulence genes and animal species. A strong relationship also exists between the occurrence of *E. faecalis* and animal species. Our study has demonstrated that pets can be considered as a reservoir of potentially pathogenic Enterococci endowed with antimicrobial resistance and virulence factors. Therefore, we cannot exclude the possibility that Enterococci isolated from dogs and cats may be responsible for opportunistic infections in humans, particularly among high-risk owners. More studies should be conducted in order to investigate the prevalence of pathogenic *Enterococcus* spp. from cattle and horses because this study did not detect the two species, and a small sample size was used.
